# Assessment of the Effectiveness of Treatment of Vascular Lesions within the Facial Skin with a Laser with a Wavelength of 532 nm Based on Photographic Diagnostics with the Use of Polarized Light

**DOI:** 10.3390/s23021010

**Published:** 2023-01-16

**Authors:** Piotr Zawodny, Wiola Malec, Kamil Gill, Karolina Skonieczna-Żydecka, Jerzy Sieńko

**Affiliations:** 1 Zawodny Esthetic Medicine Clinic, 71-047 Szczecin, Poland; 2 Department of Histology and Developmental Biology, Pomeranian Medical University in Szczecin, 71-210 Szczecin, Poland; 3 Department of Biochemical Science, Pomeranian Medical University in Szczecin, 71-460 Szczecin, Poland; 4 Department of General Surgery and Transplantation, Pomeranian Medical University in Szczecin, 70-111 Szczecin, Poland

**Keywords:** laser therapy, vascular laser, KTP laser, vascular skin lesions, aesthetic medicine, VISIA

## Abstract

Aesthetic medicine is a dynamically developing field of medicine. It has an impact not only on the improvement of the external appearance, but also on health and quality of life. Currently, vascular changes affect many patients and significantly diminish the condition of the skin. The development of modern laser therapy has contributed to the successful management of multiple skin conditions, among them vascular lesions. The aim of our study was to show the efficacy of repetitive 532 nm laser therapy in reducing vascular skin lesions located on the facial area. Observations were possible due to the implementation of System of Skin Analysis. We retrospectively analyzed the records of 120 patients (100 women and 20 men) using “VISIA” Skin Analysis System after 532 nm laser therapy. Treatment with the use of the 532 nm vascular laser turned out to be effective in reducing vascular changes in both women and men. The skin phototypes did not significantly affect the therapy efficacy. Neither the age of patients nor number of laser sessions affect therapy efficacy. 532 nm laser therapy is effective in reducing vascular skin lesions located in the face area.

## 1. Introduction

The broadly understood definition of vascular skin refers to the presence of changes on the facial skin, such as telangiectasia, paroxysmal or permanent erythema, and sometimes inflammatory eruptions. Vascular lesions also include all types of hemangioma, which are a group of benign neoplastic lesions originating from the vascular tissue. There are capillary, cavernous and mixed hemangiomas due to their structure. The latter ones are also categorized as lymphangiomas and blood hemangiomas [1,2].

In the aesthetic sense, vascular skin is the proof of high environmental exposure. The zones being first-line exponents are the face, neck and cleavage lines. Due to the tendency of skin microcirculation to dilate, these areas belong to the erythematous areas of the body (so-called blush area, flushing region) [3,4]. In a cross-sectional study it was found that older persons, males, smokers, and outdoor workers are at highest risk for telangiectasia development [5].

An important aspect characterizing the vascular skin is the visible course of somatic reactions in patients who have this type of skin. The tension of the skin vessel walls and the course of metabolic and inflammatory processes in their environment are modulated by emotions, based on the relationship between hormonal and immune stimuli, the autonomic nervous system and neurotransmitters. The rich innervation of the facial skin vessels by the post-ganglionic fibers of the sympathetic system causes a vivid vascular game in this area of the body [6,7].

Facial vascular skin is subjected to therapies aimed at vascular lesion reduction or total elimination. Treatment of vascular lesions, depending on their type, is carried out using various methods, which include electrocoagulation, Intense Pulse Light (IPL) laser treatment, sclerotherapy and surgical treatment. Electrocoagulation involves the use of an electromagnetic field or direct current to thermally close vascular lesions. Sclerotherapy is an injection procedure using a vasoconstrictor administered intravascularly. The most radical and invasive procedure is surgical excision of the vascular lesion. Only laser therapy and IPL from the above-mentioned treatments do not disturb the continuity of the skin, and at the same time, depending on the wavelength, they selectively affect hemoglobin contained in blood vessels.

IPL first evaluated in a rabbit model was found to minimize the production of a purpura and epidermal damage [8]. IPL uses a flashlamp to emit polychromatic light in a spectrum of 400–1400 nm, which gives versatility in dermatology treatment. Of note, the wavelength of light might be adjusted by applying filters. The most commonly used filters in IPL platforms used in the treatment of vascular lesions start with the wavelength range of 515 nm, 560 nm, 590 nm or have a separate range of 500–600 nm or 515–600 nm and at the same time 800–1200 nm. Wavelength, pulse duration, fluence, and spot size constitute variables affecting the IPL efficacy [9].

Monochromatic, collimated and coherent light is emitted by lasers. Lasers used in vascular therapy include: pulsed dye laser with a wavelength of 577 nm, 585 nm, 590 nm, 595 nm and a pulse duration of 0.45–1.5 ms; argon laser with a wavelength of 488 nm, 514 nm and a pulse duration of 50–200 ms; copper laser with a wavelength of 578 nm and a pulse duration of 50–200 ms; potassium-titanium-phosphate laser (KTP laser) with a wavelength of 532 nm and a pulse duration of 2–20 ms; neodymium-doped yttrium aluminum garnet laser (ND:YAG laser) with a wavelength of 1064 nm and a pulse duration of 10–60 ms; krypton laser with a wavelength of 568 nm and a pulse duration of 50–200 ms and a CO_2_ laser with a wavelength of 10,600 nm operating in the form of pulsed SP (Super Pulse), Ultra Pulse and continuous waves [10,11]. Among the above-mentioned lasers, argon, copper and krypton lasers are no longer used due to the induction of pigmentation changes (post-inflammatory hyperpigmentation) and scarring CO_2_ lasers, due to the strong absorption by H_2_O, are currently used in the treatment of cavernous angiomas and in the combined treatment of vascular changes accompanied by connective tissue hyperplasia with other lasers, such as KTP and PDL. Short-pulse lasers Q-switch (pulse duration measured in nanoseconds -ns) and ultra-short-pulse so-called picosecond pulses (pulse duration measured in picoseconds—ps) despite the 532 n/1064 nm wavelengths appropriate for vascular lesions, due to the too short pulse duration and therefore the inability to accumulate thermal energy in tissues, are not applicable in the treatment of vascular lesions. However, they are used and are great for treating pigmented lesions and removing tattoos [12,13].

Therapies based on the light source show significant effectiveness in reducing superficial vascular changes in the face area. Their action is based on selective photo thermolysis [14,15]. During the procedure, a photothermal effect is induced by the light pulse generated by the device acting on the tissue. Chromophores (oxyhemoglobin) present in the tissues, absorbing the laser light, cause an increase in temperature and the destruction of the irradiated structure [11]. When selecting the appropriate device and its parameters for a specific vascular lesion, the depth of penetration of the laser beam should be taken into account. The depth of penetration of the light beam into the tissues is influenced by such laser parameters as: the wavelength and the diameter of the light beam. The penetration and absorption of light by the skin is determined by the optical properties of the skin, which depend on absorption and scattering and are their resultant. Absorption of photons of light and its coefficient depends most on the spectrum of light, that is, the wavelength and concentration of skin chromophores. However, the increase in photon penetration depth and photothermolysis efficiency is also influenced by the spot diameter and is the result of multiple scattering in the dermis, which, due to the larger irradiation field, allows more photons to stay within the diameter of the laser beam. In contrast, with a smaller beam diameter, a larger number of photons will scatter inefficiently beyond the irradiation spot [16,17]. The wavelength, such as 532 nm, affects superficial blood vessels containing oxygenated hemoglobin–oxyhemoglobin in red, while the wavelength of 1064 nm is absorbed by deeper vessels in purple and blue. Thus, the deeper the vessel is located, the higher the wavelength should be used [11,12].

The effectiveness of selective photothermolysis depends on the third equally important parameter, which is the duration of the laser light pulse. This parameter affects the efficiency of coagulation of a given vessel or vessels and this relationship is conditioned by the thermal relaxation time of the tissues. That is, the relationship looks as follows: the smaller the vessel diameter, the shorter the pulse duration should be, while the larger the vessel diameter, the longer the pulse duration should be. Overall, the fluence—being an energy of laser light delivered per area—depends on vessel colour but also its size, depth of location and vessel pressure. At last, the laser therapy efficacy depends on the host characteristics, such as skin type and age of patients. Light skin types require shorter pulses, shorter pulse intervals and smaller fluence. Youngest patients typically have smaller vessels with less in-depth locations, and are thus more vulnerable to laser therapy [18].

The percentage of patients undergoing cosmetic procedures is increasing over time. Aesthetic medicine not only improves one’s physical health, but also contributes largely to mental and social aspects of human life. Facial vascular lesions are one of the most common reasons why patients seek medical advice in dermatology outpatient clinics. Therefore, we aimed to analyze the efficacy of commonly used, repetitive 532 nm laser therapy in reducing facial vascular lesions.

## 2. Materials and Methods

### 2.1. Patients

The effectiveness of vascular laser treatments with a wavelength of 532 nm was assessed among patients who underwent laser therapy at the Zawodny Esthetic Clinic in Szczecin in the period from September 2017 to April 2018. A group of 120 persons who were photographed using the VISIA analysis system more than once qualified for the study.

The study group consisted of 100 women and 20 men. The age of the patients was between 19 and 68 years, and the mean age was 43.34 ± 11.23 years. All these people came to the Clinic due to erythematous and vascular changes of varying severity. Most of the patients had skin erythema, telangiectasia, and inflammatory papules with the starting process of rosacea. Hemangiomas were diagnosed in 6 patients. In one patient, the angioma was cavernous, while in the remaining five patients flat hemangiomas were detected.

### 2.2. Therapy

The laser (potassium-titanyl-phosphate; KTP; Cutera Company laser Excel V, Brisbane, CA, USA) that was used to eliminate vascular changes in patients in the research group was a vascular laser with double-wavelength technology (532/1064 nm). This system enables the selection of parameters according to the needs of a given patient and the treatment of all vascular diseases, from minor telangiectasias to major vascular changes. This device, thanks to a large light spot, the size of which ranges from 1.5 to 12 mm, has the ability to deeply penetrate the skin layers. The 532 nm laser beam is strongly absorbed by oxyhemoglobin. In our patients from the study group, we used a light beam with a length of 532 nm, spot diameter in the range of 4–12 mm and pulse duration in the range of 6–10 ms. As a result, the process of blood photocoagulation and vessel damage occurs, which ultimately leads to its fibrosis and spontaneous atrophy. This process occurs without breaking the epidermis and damaging the surrounding tissues.

### 2.3. Efficacy

To evaluate the efficacy of laser therapy, the VISIA Skin Analysis System determining the condition of the skin objectively and reproducibly using numerical values was used. The device is a photographic booth, which transmits data to a connected computer with special VISIA software. This system uses the RBX (Red/Brown Subsurface Analysis) technology, which determines the number of superficial blood vessels exceeding the established norm (from now on called: red areas number and allows the analysis of the condition of the superficial blood vessels (from now on, called the VISIA score). The standard is determined by the VISIA database, on the basis of which it is possible to compare the skin analysis of a given patient to other people of the same age and skin type [19]. The outcomes we assessed were then: the red area number and VISIA score at baseline and after a series of laser sessions. Moreover, a delta score was (endpoint score minus baseline score) was calculated for analyses.

### 2.4. Statistical Analyses

The distribution of continuous variables was assessed using the Shapiro-Wilk test, assuming compliance at the significance level of *p* > 0.05. The non-parametric Mann-Whitney U test or Kruskal-Wallis as appropriate were used for comparing data between independent groups. For the analysis of the obtained effects of laser therapy, the results were compared in a within subject manner using the Wilcoxon test. Linear relationships between the studied variables were verified by calculating the Spearman rank correlation coefficient^®^. For all tests used, statistical significance was considered at *p* < 0.05. Statistical analysis was conducted using MedCalc software (version 20.110; Ostend, Belgium).

## 3. Results

Among patients analysed in the present study, there were 80 people (*n* = 80) who had skin phototype I (median age: 41 years, range 21–68 years old) and 31 patients (*n* = 31)—phototype II (median age: 42 years old, range 19–67 years). Phototype III was established in 9 participants (median age 50 years, range 39–68). These differences were not statistically significant (*p* = 0.11). At least 1 (max 6) laser procedure was utilized (IQR: 1–2). The comparison of Red No. areas and VISIA score in a within-subject design proved a significant reduction in both of these parameters post-laser therapy. The results are shown in Table 1.

**Figure 1:**
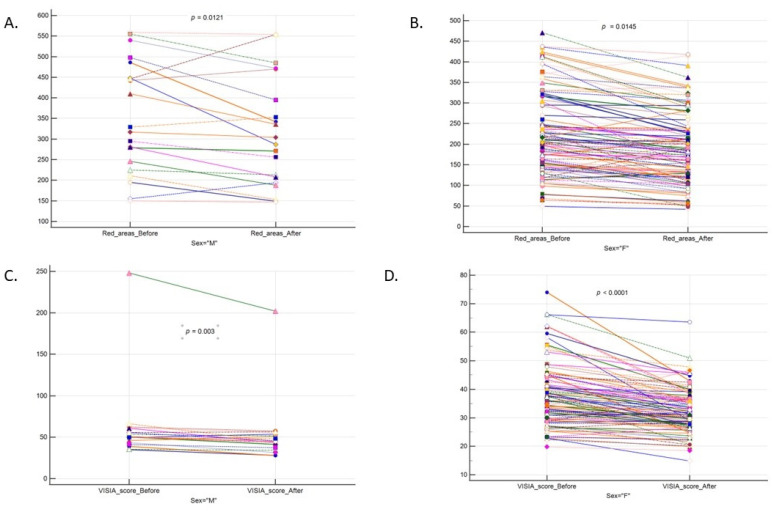
Laser therapy efficacy regarding red number areas in (**A**). Males, (**B**). Females and VISIA score in (**C**). Males and (**D**). Females. Note, VISIA score in men after excluding an outlier; *p* = 0.0005.

This was also confirmed when analyzing the results separately for females and males as shown in Figure 1. When we excluded an outlier (*n* = 1; clearly visible in Figure 1C) in the men subgroup, the results remained significant (median before: 50.038; median after: 44.92; paired difference −5.422; *p* = 0.0005).

**Figure 2:**
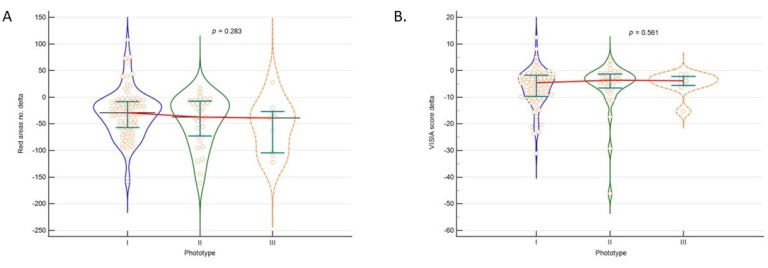
Treatment efficacy in regard to (**A**). Red area number and (**B**). VISI score regarding skin phototype.

Laser treatment efficacy expressed as delta scores for both outcomes that were analysed were not significantly influenced by skin phototype (*p* > 0.05; Figure 2).

There were some correlations found between the tested parameters and number of laser procedures and age of participants. These were, however, present only in a whole group and in females, not in males. We found that the VISIA score evaluated before and after the laser procedure was positively correlated to the age of participants. In the case of VISIA score at the endpoint, the result was positively correlated to the number of laser interventions as well. We also found a statistical tendency toward a higher red areas number at endpoint with age (*p* = 0.06). Results can be found in Table 2.

## 4. Discussion

Mild vascular lesions in the facial area are a common dermatological issue which might cause low self-esteem and even diminish quality of life, as evidenced by means of Dermatology Life Quality Index in patients suffering from rosacea [20]. With the advent of laser techniques, treatment of these lesions is fast but depends on the laser wavelength thus depth of penetration [21,22].

In our study we retrospectively analysed 120 patients with facial redness of various types who underwent KTP laser treatment. We here confirm that the efficacy is neither dependent on skin phototype nor the gender and age of participants. Additionally, number of laser sessions is not a matter of significance. Overall, KTP laser turned out to be effective in the treatment of vascular lesions in the facial skin. Similarly, the effectiveness of the 532 nm beam in the treatment of the above-mentioned changes were observed by Carniol et al. [23]. The authors indicated a very good telangiectasia response to treatment with a laser with a wavelength of 532 nm, however larger and deeper lesions needed to be treated with a 940 nm laser. Good results of using the KTP laser are also described by Cassuto et al., [24] who in their reports indicated an improvement in the condition of blood vessels within the facial skin at the level of 75–100% in almost 94.0% of people who underwent only one KTP laser treatment. A second session was conducted for the rest of the study group. On the other hand, Uebelhoer et al. [25] when assessing the effectiveness of vessel-sealing with the 532 nm beam, showed an improvement of 62% after one treatment which elevated by 23% three weeks after the third treatment. The authors also compared the effectiveness of the KTP and PDL lasers in the same patients. The KTP laser was shown to be more effective. In a study by Becher et al. [26] the authors evaluated the efficacy of the KTP laser in 647 patients. The so-called “clearance” or “marked improvement” was reported by 77.6% of patients. However, only one-third of patients with facial, rosaceal, erythema improved with the treatment as compared to baseline probably due to the long pulse of the KTP being unable to match the thermal relaxation time of the tiny vessels. Keaney et al. [21] who conducted research in patients with Erythematous Surgical Scars compared the efficacy of 532 nm KTP and 595 nm PDL systems and found no significant differences adopting blinded photo assessments, satisfaction evaluation, scar symptoms, and intraoperative pain scores.

Until recently [27], the standard of treatment for vascular skin lesions was the PDL 595 nm laser. Indeed, the laser provides good penetration into the dermis, however high costs and the risk of rupture of vessels, especially superficial ones, make it unattractive. Overall, as elegantly discussed elsewhere [13,28,29,30], many different lasers can be utilized to successfully treat vascular lesions, however a 532 nm laser with the advent of efficient cutaneous cooling and lengthening of pulse duration makes this particular one less painful with a smaller probability of developing purpura compared to other ones. Indeed, a 532 nm laser has a higher absorption coefficient for oxyhemoglobin as compared to a 595 nm pulse dyed laser [25]. Importantly in clinical practice, pigmentation and even small wrinkles can be treated with such a wavelength, making this an optimal choice also from an economic point of view [30]. As expected, we found no relationship between KTP laser efficacy and skin type. In our cohort, patients had 1–3 skin phototypes only. As skin type number elevates melanin content, melanosome counts and size, the reactive fibroblasts number increase. Melanin acts as a chromophore-absorbing laser energy; thus, in darkly pigmented skin the risk of epidermal injury increases [31].

The results obtained with the use of the VISIA Skin Analysis System showed a significant improvement in skin quality in all patients, regardless of age and gender. Additionally, Cadavid et al. [32], who analyzed the effectiveness of laser therapy with a laser with a wavelength of 532 nm, assessed the effectiveness of the treatments as highly effective, regardless of gender. The group studied by the authors consisted of 93 patients, including 57 women and 36 men. The authors assessed laser therapy of vascular lesions as effective and safe. The effectiveness of therapy in cases of both genders and regardless of age was also indicated by Becher et al., where the group of women who took part in the study was 486 people. There were 161 men in the study. The efficacy was similar between genders [26].

There are no unequivocal data in the literature on the physical parameters of the laser beam used in the treatment of vascular skin lesions. It is known from numerous scientific reports that the wavelength best absorbed by hemoglobin is the one characterized by shallow penetration into the tissue (range 530–590 nm). The own experience and reports of other authors show that the length of the laser pulse should be adjusted to the diameter of the vessel, taking into account the thermal relaxation time of the target structure. Accordingly, vessels with a relatively small diameter will require shorter pulses and vessels with a larger diameter will require a correspondingly longer pulse [29]. The availability on the market of devices emitting the wavelength of 532 nm is high. Comparing efficacy indices of these instruments is somehow challenging as different lesions were treated and various parameters of efficacy assessment were reported by the researchers. For instance, in a study by Keaney et al. [21], a physician global assessment (PGA), including the Vancouver scar scale, was utilized to test the efficacy. Carniol et al. [23] used visible change in the appearance of the vessel, whilst Cassuto et al. [24] tested the efficacy evaluating percentage of the telangiectasias using a milli-metric transparent grid. Uebelhoer et al. [25] asked a blinded investigator to evaluate the percent resolution of baseline diffuse telangiectasia. Becher et al. [26] also subjectively assessed the efficacy using photographs. These all state, that the objective assessment of the treated lesions is critical in laser treatment of vascular lesions. Nevertheless, as for the available evidence, the efficacy of the KTP laser in treatment of vascular lesions is high and possesses minimal risk of harmful effects. 

Our study has some limitations. We did not evaluate adverse effects, however we confirm that no serious injuries were noted. Although therapies based on selective photothermolysis effects are non-invasive in nature, this parameter is crucial in human studies [13]. Unfortunately, we did not measure patients’ satisfaction as well. As pointed out in multiple studies, patient well-being defined as “emotional, physical, social, and functional dimensions related to health” should be evaluated in medical research and care [33].

## 5. Conclusions

The use of the 532 nm wavelength laser turned out to be effective in reducing vascular lesions, because a significant reduction in the number of red areas and VISIA score was observed in the entire population covered by the study. This was also confirmed after excluding an outlier. The therapy has been successful in both women and men. In the study group, the described method of therapy turned out to be effective for all of the considered skin phototypes. The skin phototype of the study participants did not significantly affect the number of red areas and the results, which were verified both before and after the procedure.

## Figures and Tables

**Table 1:** Comparison of the number of red areas and the VISIA score of the assessment before and after the treatment in the whole study group.

Variable	RED Areas Number		VISIA Score	
Time Point	Before	After	Before	After
Sample size	120		120	
Lowest value	49	42	19.808	14.844
Highest value	559	554	248.11	201.942
Median	216–	191.5	38.319	32.3175
95% CI for the median	201.3581–241.4279	173.7860–212.0000	35.8655–40.6380	31.1233–34.7875
Interquartile range	155.0000–322.5000	130.5000–281.5000	31.7955–46.2155	27.9300–39.7900
Hodges-Lehmann median difference	−34		−5.0308	
95% Confidence interval	−42.5000–−26.0000		−6.1065–−3.9755	
Two-tailed probability	*p* < 0.0001		*p* < 0.0001	

**Table 2:** Correlation analysis between studied parameters and number of laser procedures and age of persons regarding sex.

		Whole Group		Men		Women	
		Number_of_Procedures	Age	Number_of_Procedures	Age	Number_of_Procedures	Age
Red areas no. delta	r	0.112	0.12	0.244	0.156	0.094	0.137
	*p*	0.2215	0.1926	0.299	0.5115	0.3506	0.1742
	*n*	120	120	20	20	100	100
VISIA score delta	r	−0.006	−0.076	−0.034	−0.209	−0.024	−0.014
	*p*	0.9474	0.4098	0.8873	0.3757	0.8158	0.8935
	*n*	120	120	20	20	100	100

## Data Availability

The data presented in this study are available on request from the corresponding author.
